# Using XRD to Assess the Strength of Fly-Ash- and Metakaolin-Based Geopolymers

**DOI:** 10.3390/ma18092093

**Published:** 2025-05-02

**Authors:** Arie van Riessen, Evan Jamieson, Hendrik Gildenhuys, Ramon Skane, Jarrad Allery

**Affiliations:** Future Battery Industries CRC, John de Laeter Research Centre, Curtin University, Perth, WA 6845, Australia; evan.jamieson@curtin.edu.au (E.J.); hendrik.gildenhuys@curtin.edu.au (H.G.); ramon.skane@postgrad.curtin.edu.au (R.S.); jarrad.allery@curtin.edu.au (J.A.)

**Keywords:** geopolymer, XRD, strength, amorphous hump

## Abstract

Compressive strength testing is usually the first test applied to alkali-activated materials or geopolymers after their manufacture to gauge the success of the selected formulation. If the compressive strength is found to be acceptable, then a raft of other tests can be applied to assess the suitability of the geopolymer for its anticipated application. It is proposed that a rapid X-ray diffraction (XRD) test can provide an indication of the strength of geopolymers via the measurement of the difference between the amorphous precursor peak position and the position of the amorphous peak of the geopolymer material. The proposed XRD method provides complementary data to mechanical strength testing and provides evidence that a geopolymer has formed.

## 1. Introduction

Alkali-activated materials (AAMs) or geopolymers are X-ray amorphous binders that have the potential to replace ordinary Portland cement (OPC) due to their lower carbon footprint [1,2,3,4] and superior mechanical and chemical properties [5,6,7]. The solid aluminosilicate precursors for geopolymers often include metakaolin [8,9,10,11,12,13,14], coal-fired power station fly ash [15,16,17,18,19,20,21], or ground granulated steel slag [22,23,24,25,26]. Some of these materials are industrial by-products that are often disposed of in tailing dams or repositories and can now be utilised to make geopolymers, thus contributing to the circular economy [27,28].

Several analytical techniques are used to evaluate precursors and their subsequent geopolymers. For instance, X-ray fluorescence (XRF) is used for assessing the overall elemental composition, X-ray diffraction (XRD) is used for crystalline phase analysis and for determining the amount of amorphous material [29], scanning electron microscopy and energy dispersive X-ray analysis are used for size and composition analysis, etc. However, XRD is fast becoming an essential technique in geopolymer formulation development and quality control. For example, fly ash is a variable product whose composition changes as the coal source changes and as the power station’s burning conditions change [30]. To cater for the changes in fly ash, it is essential to characterise the ash regularly for consistent quality control. This can be achieved by conducting XRF analysis combined with XRD analysis of the fly ash to determine the amount of amorphous material and its composition [31]. This information enables specific alkali activator solutions to be designed for geopolymers with targeted Si/Al and Na/Al. Thus, the role of XRD in formulation design is essential for reagent quality control and product quality assurance. Once the geopolymer has been manufactured, XRD analytics can also be used to assess the success of the formulation via determining the extent of dissolution of the precursor and the amount of geopolymer formed based on the amorphous hump characteristics.

Davidovits [32,33] recognised the importance of geopolymeric amorphous humps from X-ray diffractograms and measured their positions for several different metakaolin-based formulations, although no clear trend was identified. Subaer [34] found that the amorphous hump shifted to higher 2Ɵ values as the Si/Al ratio decreased but did not quantify the shift. Chen-Tan [35] found that the amorphous hump in fly-ash-based geopolymerswas about 6° 2θ (for CuKα radiation) higher than the amorphous hump for the precursor fly ash. Williams et al. [29] used the amorphous hump via a detailed experiment to determine the amount of unreacted metakaolin. Williams et al. [29] also recognised that a method for the qualitative analysis of the degree of geopolymer reaction is to measure how far the amorphous hump shifts to a higher 2θ. Aluminosilicates such as metakaolin exhibit a characteristic broad XRD peak at around 22° (2θ), indicative of their short-range atomic ordering [36,37]. Upon dissolution, metakaolin releases monomeric or oligomeric silicate and aluminate species into the solution [38]. These species undergo transport and reorganisation, eventually condensing via M–O–M (M = Si, Al) bond formation, leading to gelation [39]. As the gel hardens, a solid network forms through the combination of Si and Al tetrahedra, with Na+ ions balancing the charge of the Al tetrahedra [39,40]. This restructured aluminosilicate displays altered nearest-neighbour bond lengths, manifested as a shift in the amorphous XRD peak to higher 2θ values [41]. The Si/Al ratio of the resulting geopolymer significantly influences the ordering of the Si and Al tetrahedra, thereby affecting its density and mechanical strength [41,42].

Careful assessment of geopolymer precursor XRD patterns from metakaolin and fly ash reveal two clear features: the first is a series of sharp reflections from the crystalline phases, and the second is a broad hump from the amorphous material. Typically, crystalline phases do not contribute to geopolymerisation and are present in the final product as a fine aggregate or filler [43]. However, it is important to know the abundance of the crystalline phases for formulation development. For instance, in metakaolin made from high-grade kaolin, quartz and anatase might be present at trace levels [44], while in fly ash, the crystalline phases (quartz, mullite, and iron oxide) could be 50 wt% or more [6]. For fly ash, the large number of crystalline reflections often obscures the amorphous hump, while for metakaolin, it is more obvious. Due to the broad nature of the amorphous hump compared with crystalline reflections, it is often ignored as a possible source of additional information about precursor dissolution and geopolymerisation. This paper presents a method to extract considerable further information from the amorphous hump for geopolymer characterisation and correlations with compressive strength.

## 2. Experimental Details

Two kaolin (Prestige kaolin, Sibelco, McIntyre, USA and K999, WA Kaolin Ltd., Wickepin, Australia) and two fly ash samples (Synergy power station, sampled in 2022 and 2023, Muja, Australia) were selected for this experiment. The kaolin samples were chosen because they provide a clearer definition of the amorphous hump in their XRD patterns after calcining at 750 °C for 8 h, while the microstructurally more complex fly ash samples generated from burning subbituminous coal are more likely to be made into commercial geopolymers. X-ray fluorescence (XRF) was used to obtain the elemental composition of the samples and their bulk Si/Al values (Table 1). The XRF and Loss on Ignition (LOI) were determined by a commercial laboratory (Intertek, Perth, Western Australia) using fusion beads and calibrated with relevant certified standards. Quantitative XRD provided the abundance of the crystalline phases and the amorphous material (Table 2). It should be noted that for the fly ash samples, two quartz components were modelled [29]. Quartz 1 was modelled with a large crystallite size and represents large quartz particles that were separate from the fly ash spheres. Quartz 2 was modelled with a small crystallite size and was present within the fly ash spheres as exsolved quartz resulting from the breakdown of kaolin into quartz and mullite during the burning of coal [45]. The composition of the amorphous materials was calculated by subtracting the elemental component of all the crystalline phases from the bulk XRF elemental data (Table 3).

The amount of amorphous material and its composition were used to make geopolymers with a range of Si/Al and Na/Al values (Table 4). This necessitated using NaOH, NaOH + Na silicate, and NaOH + Na aluminate to achieve the targeted range of Si/Al values (Table 4). Both the fly ash geopolymer systems were formulated with a Na/Al ratio of 1.0, and both metakaolin geopolymers were made with varying Na/Al ratios to induce a wider variation in the resulting compressive strength values. The water content (H/Si ratio) was selected to ensure sufficient workability across the range of targeted Si/Al ratios. The total H_2_O *w*/*w*% values were MK 1 = 29.1%, MK 2 = 35.0%, FA 1 = 18.0%, and FA 2 = 15.0 wt.%.

The following mixing, casting, and curing processes were adopted.

The alkali activator was prepared following a controlled sequence, where deionised water was first added to a mixing vessel, followed by the gradual addition of NaOH pellets (≥99 wt.%, Rowe Scientific). This process caused an exothermic reaction, leading to a rise in temperature, which peaked at Tmax. Once the temperature dropped to 90% of Tmax, either sodium silicate (Coogee, H-grade, SiO_2_/Na_2_O = 2.32) or sodium aluminate (Coogee, Al_2_O_3_/Na_2_O = 0.745) was introduced. This moment was designated as time = 0, and the activator was allowed to mix for 30 min before use [46]. If no additional water was used, NaOH was dissolved directly in either the silicate or aluminate solution. The solid aluminosilicate precursor was then added to the liquid activator and mixed for 5 min in a planetary mixer (Spar, SP-800A). The resulting paste was placed in 25 mL polypropylene vials, followed by 30 s of vibration. The vials were sealed prior to placing in an oven for curing at 70 °C for 21 h. After curing, the samples were demoulded and sealed in plastic bags and stored for 24 h. Approximately 48 h after mixing, the samples were tested for their compressive strengths based on ASTM C39 [47]. Fragments from the compressive strength test were used for the XRD and SEM analyses.

For the SEM analysis, precursor samples were placed on aluminum stubs using carbon tape. The samples were then coated with 10 nm of carbon using a Cressington carbon evaporative coater. Geopolymer fragments with flat surfaces were selected and embedded in 25 mm diameter × 10 mm high epoxy mounts using a Struers CitoVac machine. The mounts were then polished with ethanol using a Struers Tegramin automatic grinding and polishing machine, finishing with 1 µm diamond suspensions. The polished mounts were then coated with 10 nm of carbon using a Cressington carbon evaporative coater. The 25 mm polished mounts were placed in a top-mount reference holder to ensure a consistent working distance between samples.

For the XRD analysis, both precursor and geopolymer samples were prepared similarly, except that the geopolymer samples were first ring-milled to reduce large fragments into a fine powder. The powders were then micronised in a McCrone canister with 8 mL of laboratory-grade ethanol and corundum grinding media for 5 min. For the precursor samples, an 11 wt.% corundum internal standard was added to enable quantitative XRD to be undertaken. The resulting suspension was poured into a polypropylene dish, dried at 40 °C for 24 h, and packed into a PMMA sample holder.

XRD patterns were collected using a Bruker D8A diffractometer operating at 40 kV and 40 mA using Cu Kα radiation. A LynxEye detector (Bruker, Billerica, MA, USA) with an opening of 2.938° was used. The primary and secondary slits were 2.5°, and the divergence slit was 0.3°, and the anti-scatter slit was 18°. The data were collected using a 2θ step size of 0.015°, 2θ range of 5–120°, and time/step of 0.75 s. Phase identification was carried out with a DIFFRAC.EVA (V 6.0) and the crystalline phases were identified via the PDF 5+ database.

**Figure 1:**
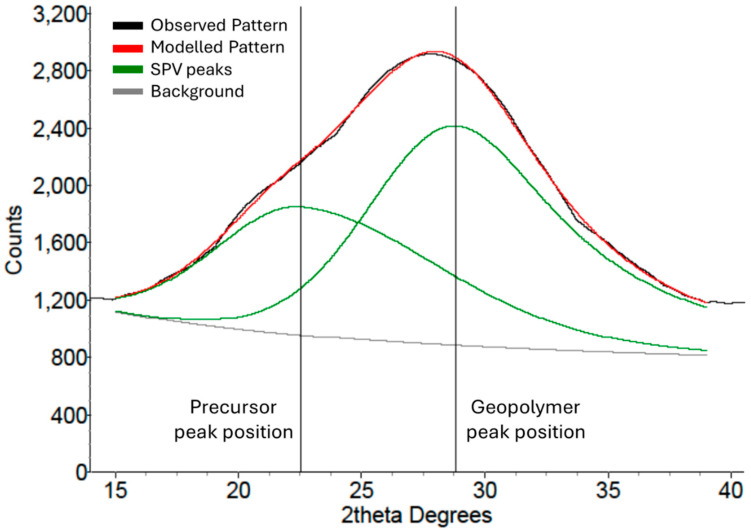
A schematic of the precursor peak position and the resulting geopolymer peak positions as modelled for the metakaolin geopolymer system in TOPAS.

The peak shift in the amorphous hump in the diffraction patterns from the precursor and geopolymers was analysed using the DIFFRAC.EVA and TOPAS (V 5.0) software through the following steps:

1. Eva was used to model and separate the background data (including amorphous humps), thus removing crystalline peaks, and exported to TOPAS.
2. In TOPAS, the precursor amorphous hump was fitted with a custom peak using a Split Pseudo-Voigt (SPV) peak profile with only the position and full width at half maximum parameters set to refine, and this centroid value was recorded.
3. The geopolymer XRD pattern was loaded into TOPAS, and two SPV peaks were fitted, one fixed at the previously recorded precursor peak position and the other refined for the geopolymer peak position.
4. The peak shift was calculated as the difference between the geopolymer peak position and the precursor peak position (Figure 1).

## 3. Results and Discussion

### 3.1. Precursors

#### 3.1.1. Scanning Electron Microscopy

**Figure 2:**
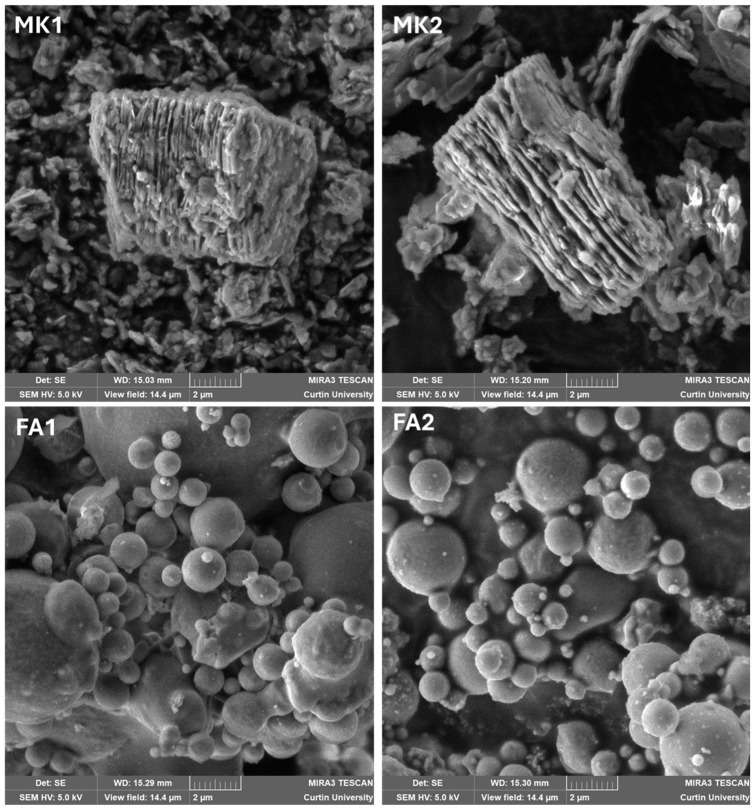
SEM images of the four precursors showing the dramatic difference in morphology between the metakaolin and fly ash.

Figure 2 shows SEM images of the four precursors. The difference in the morphology is obvious, with the metakaolin revealing its bookend structure, creating a high water demand when adding alkali. The fly ash samples exhibited the presence of spheres, where most of the amorphous material resided. The fly ash morphology enabled the water content to be reduced when mixing with alkali. As the fly ash used in this project only had approximately 50 wt.% of amorphous material, there was a lot of unreacted ash that was distributed throughout the resulting geopolymer as fine aggregate or filler.

#### 3.1.2. Particle Size Distribution

**Figure 3:**
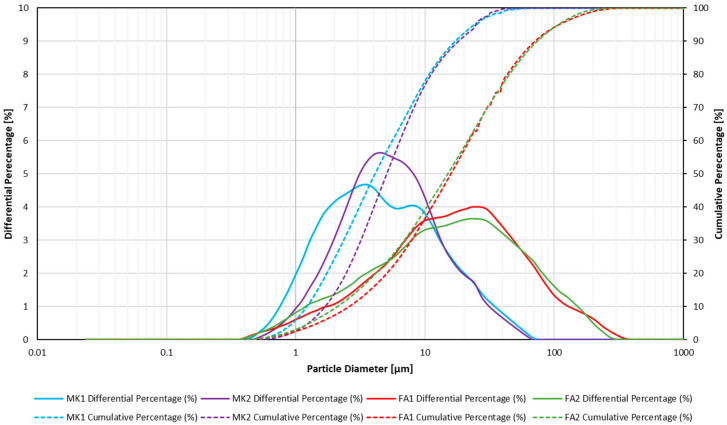
Particle size distribution of the four precursors.

Figure 3 shows the particle size distribution of the four precursors. The two fly ash samples had a similar particle size distribution, with a broad spread centred at approximately 15 µm. The broad range of particle sizes was made up of large quartz grains and carbon flakes at the large end (≥100 µm), with a wide spread of fly ash spheres right down to ≤1 µm. While quartz does not play a role in geopolymerisation, carbon (unburnt coal and activated carbon) can be detrimental to the process of geopolymerisation [48].

The metakaolin samples were also found to be similar to each other, with MK1 having a slightly finer fraction. Overall, the metakaolin samples exhibited a smaller size fraction than the fly ash samples. The combination of a smaller particle size and a platy morphology resulted in a higher water demand for the metakaolin precursors compared to the fly ash precursors.

#### 3.1.3. X-Ray Diffraction

**Figure 4:**
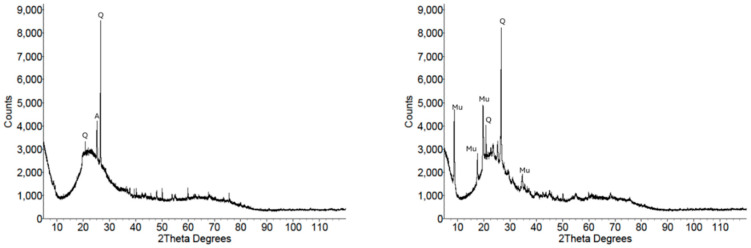
XRD patterns of precursors MK1 (**left**) and MK2 (**right**), highlighting the amorphous hump and sharp reflections from the impurity phases. Q = quartz, A = anatase, Mu = muscovite.

Figure 4 shows the XRD patterns of the two metakaolin samples. Both patterns exhibited the characteristic asymmetric amorphous hump at approximately 23° 2θ, with the crystalline impurity phases shown by the sharp reflections superimposed on the background and amorphous hump. MK1 was found to have minor impurity phases of quartz and anatase, while MK2 had crystalline impurity phases of quartz and muscovite (Table 2).

**Figure 5:**
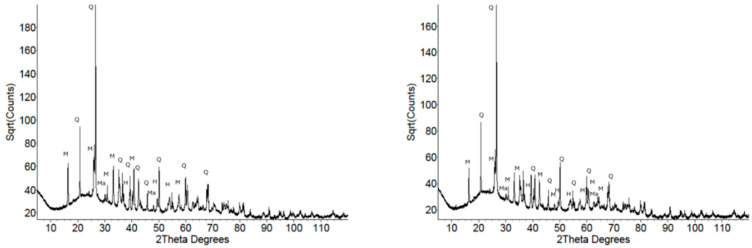
XRD patterns of precursors FA1 (**left**) and FA2 (**right**). The amorphous hump is clearly visible but subdued relative to metakaolin due to the presence of more crystalline materials. Q = quartz, M = mullite, H = hematite, Ma = magnetite.

Figure 5 shows the XRD patterns of the two fly ash samples. It is immediately apparent that the amorphous hump was more subdued, and there were many more crystalline reflections present arising from mullite, quartz, hematite, and magnetite. The measured impurity phases in the metakaolin samples amounted to 3–4 wt.% compared to 48 wt.% for the fly ash samples.

### 3.2. Geopolymers

**Figure 6:**
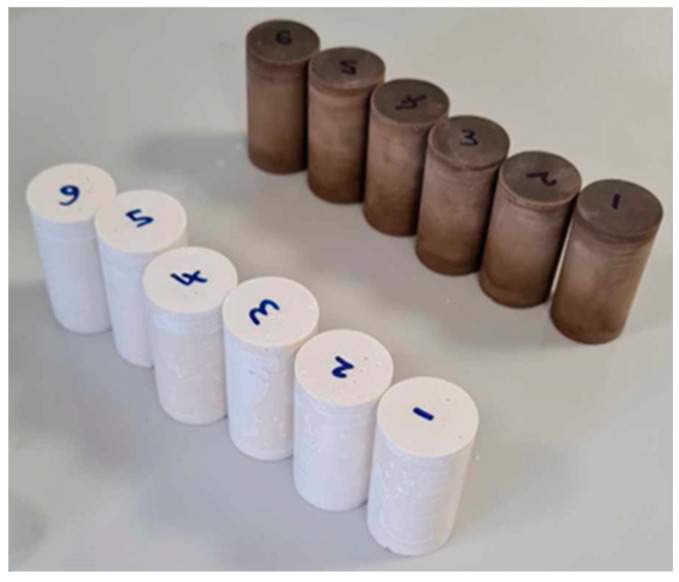
Geopolymer cylinders made from metakaolin (light) and fly ash (dark) precursors.

Figure 6 shows the geopolymer cylinders, with the lighter-coloured ones made with metakaolin and the darker ones with fly ash.

#### 3.2.1. Scanning Electron Microscopy

**Figure 7:**
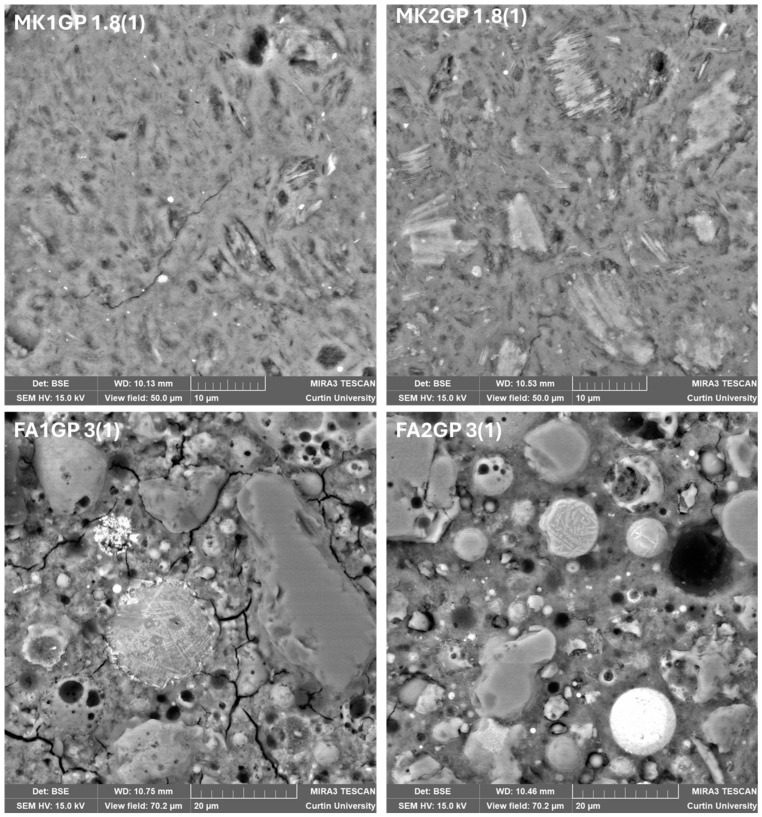
SEM images showing the microstructure of the geopolymer samples. The fly ash images were taken at a lower magnification to enable the complex microstructure to be captured. The numbering sequence on each image indicates the Si/Al (Na/Al) ratio.

Figure 7 shows SEM images of the microstructure of the geopolymer samples. The metakaolin-based geopolymer showed a dense geopolymer matrix interspersed with unreacted metakaolin (lighter colour) along with some cracks and pores. Combining X-ray elemental analysis (EDS) with imaging enabled the Si/Al and Na/Al ratios of the geopolymer matrix to be measured and compared with target values. The close alignment between the measured and target ratios confirmed the chosen mixed-design methodology to be correct, with adequate mixing and satisfactory dissolution and condensation. The presence of some unreacted metakaolin highlights the demanding nature of the geopolymer manufacturing process. However, there is no disadvantage to having a mixed matrix of geopolymer and unreacted metakaolin.

As anticipated, the fly-ash-based geopolymer had a more complex microstructure, revealing quartz, partially reacted spheres, iron oxide (bright spheres), and mullite all bound together with the geopolymer. Elemental analysis is more challenging for fly-ash-based geopolymers as it is difficult to identify large enough regions to conduct EDS without encroaching on neighbouring phases.

#### 3.2.2. X-Ray Diffraction

**Figure 8:**
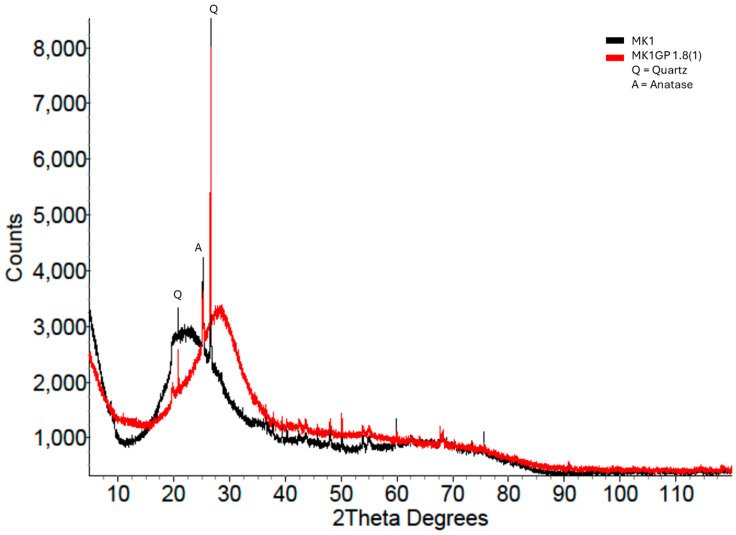
XRD pattern of MK1 geopolymer (red) overlaid on the MK1 precursor pattern (black).

**Figure 9:**
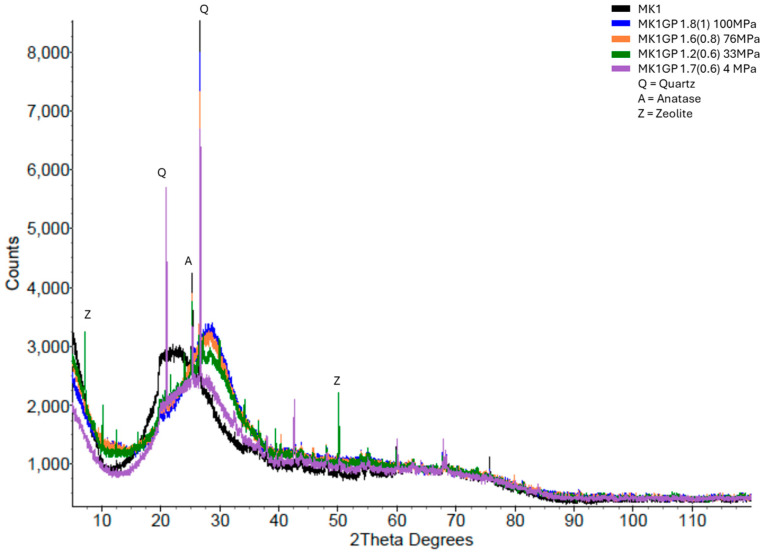
XRD patterns of four MK1 geopolymer XRD patterns overlaid on the MK1 precursor pattern.

As described in Section 2 (Experimental Details), this paper introduces a new method, based on XRD, for estimating the strength of a geopolymer by measuring the difference in the amorphous peak position between the precursor and geopolymer. Figure 8 shows the MK1 geopolymer’s XRD pattern overlaid on the MK1 precursor’s pattern. There was a clear separation between the two patterns that could be measured and that was related to the compressive strength of the geopolymer. Several MK1 geopolymer XRD patterns are overlaid on the MK1 precursor in Figure 9, showing obvious differences in both the amorphous hump position and height. The blue-coloured pattern indicates a high-strength geopolymer (100 MPa), and the purple pattern indicates a low-strength geopolymer (4 MPa). The considerably significant visual differences can be quantified as differences in the amorphous peak position (compared to the precursor) and compared to the compressive strength.

**Figure 10:**
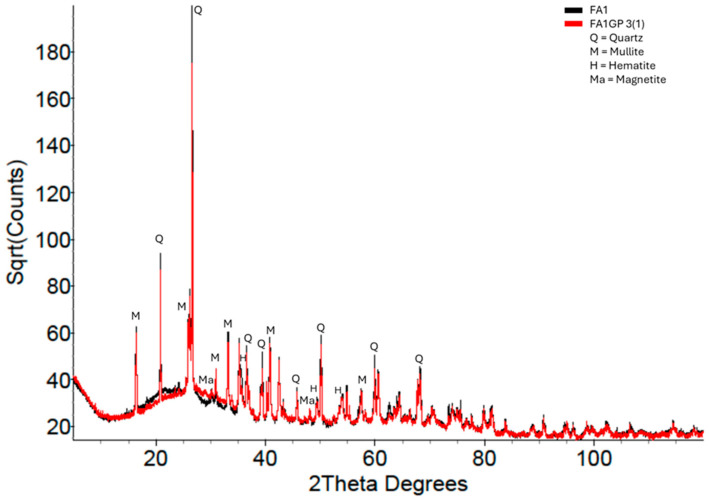
FA1 geopolymer XRD pattern overlaid on the FA1 pattern.

**Figure 11:**
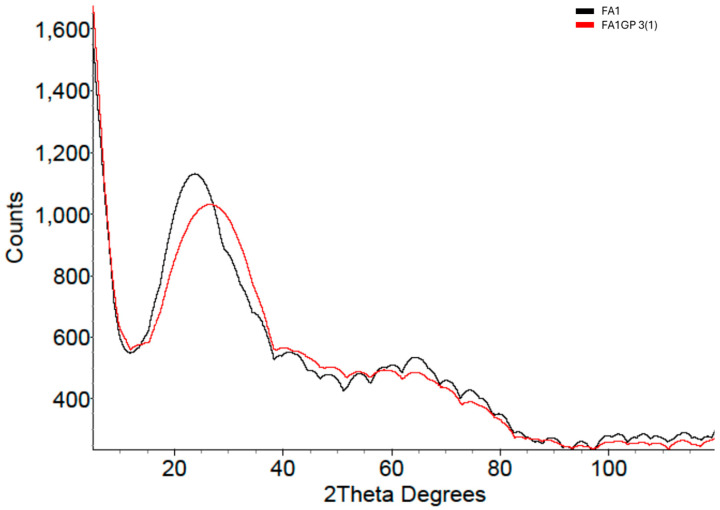
XRD patterns of FA1 precursor and FA1 geopolymer with crystalline reflections stripped, leaving only the amorphous component.

Figure 10 shows the XRD patterns of the FA1 precursor and FA1 geopolymer. Identifying the difference between the precursor and geopolymer was much more difficult, mainly due to the presence of numerous crystalline reflections. Nevertheless, stripping away the crystalline reflections emphasised the amorphous hump differences such that measurements could be made (Figure 11).

**Figure 12:**
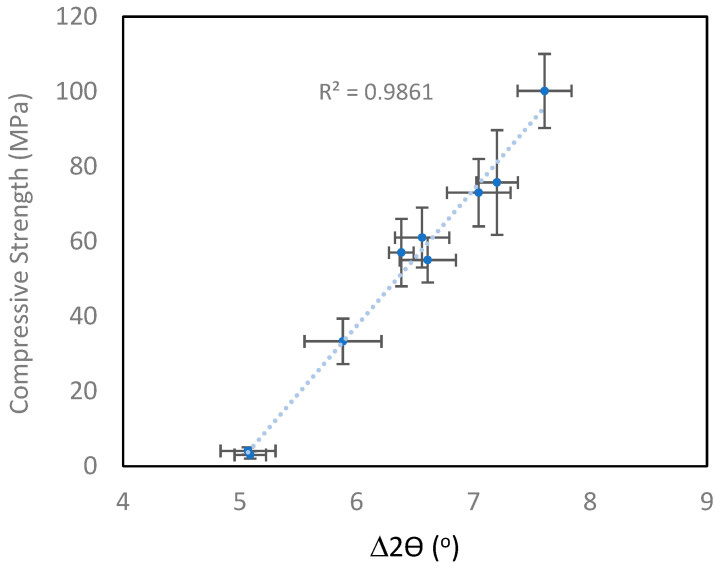
Compressive strength versus amorphous hump difference (∆2θ(°)) for two different metakaolin-based geopolymers. Error bars represent two standard deviations.

**Figure 13:**
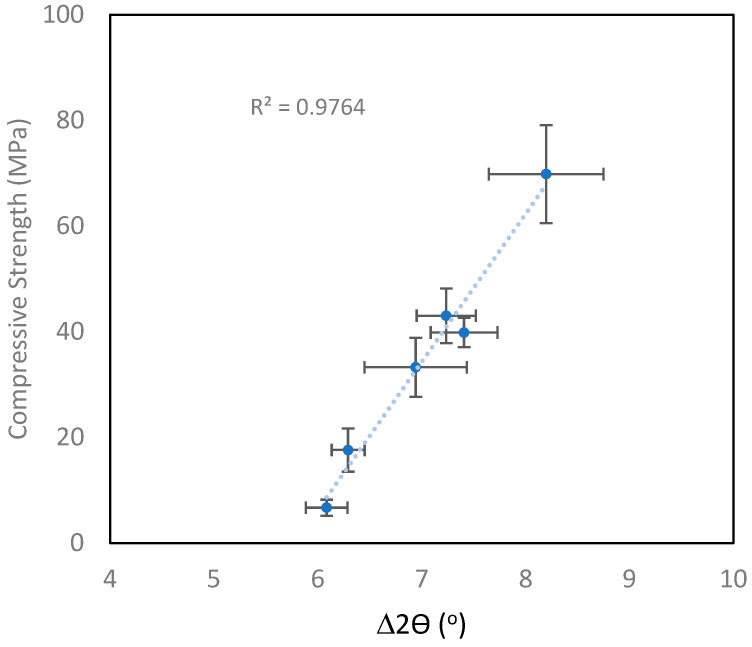
Compressive strength versus amorphous hump difference (∆2θ (°)) for two different fly-ash-based geopolymers. Error bars represent two standard deviations.

Table 5 shows the compressive strength of the four sets of geopolymer samples and the measured difference between the amorphous hump of the precursor and geopolymer. To demonstrate the versatility of the method, the MK1 and MK2 geopolymer results (Figure 12) were combined, as were the FA1 and FA2 geopolymer results (Figure 13). It is immediately apparent that there was a strong linear correlation between the strength and amorphous hump shift for both the metakaolin and fly ash sample sets. The emphatic relationship between the strength and amorphous hump shift provides an objective method for assessing the quality of geopolymers. The method is versatile and robust, as it worked for both the fly ash and metakaolin precursors. In four of the geopolymer samples (Table 5), zeolite was found to be present in the microstructure, but this did not impede the use of the peak shift method.

It should be noted that geopolymers with a range of different strengths were tested to demonstrate that the method has a wide dynamic strength range: for the fly ash geopolymer, the range was from 7 to 70 MPa, and for metakaolin geopolymer, the range was from 3 to 100 MPa.

## 4. Conclusions

A new method was developed that directly relates the strength of a geopolymer to the XRD amorphous hump shift between the precursor and geopolymer. The method is objective and robust and provides a straightforward approach to assessing geopolymer quality and strength. Previously, Williams et al. [29] demonstrated that XRD methodology and SEM techniques could be used to assess the amount of unreacted MK. Although the level of unreacted metakaolin is linked to the compressive strength of the geopolymer matrix, the Si/Al ratio of the resultant geopolymer matrix plays an important role as well. In addition, the porosity also impacts the strength, contributing to a complex mix of factors contributing to the strength. The amorphous hump shift method described here captures a wide range of microstructural effects and encapsulates them into one measurement relating directly to strength. A quantifiable XRD technique is now available that can enable the confirmation of geopolymerisation.

## Figures and Tables

**Table 1:** Oxide compositions of the metakaolin (MK) and fly ash (FA) samples as determined by XRF (wt.%).

Analyte	MK1	MK2	FA1	FA2
Al_2_O_3_	42.32	42.58	29.72	25.74
BaO	0.02	0.02	0.30	0.20
CaO	0.10	0.01	1.00	0.67
Cr_2_O_3_	X	X	0.02	0.02
Fe_2_O_3_	0.96	0.46	8.87	8.06
K_2_O	0.26	1.60	0.50	0.46
MgO	0.19	0.26	0.66	0.80
MnO	X	X	0.05	0.05
Na_2_O	0.07	0.05	0.22	0.24
P_2_O_5_	0.01	0.01	1.23	0.38
SO_3_	0.03	X	0.26	0.18
SiO_2_	53.30	53.35	53.94	60.42
TiO_2_	1.15	0.52	1.83	1.85
LOI (1000 °C)	1.49	0.88	1.17	0.74
Total	99.93	99.74	99.94	99.86
Si/Al (wt.%)	1.26	1.25	1.81	2.35
Molar Si/Al	1.36	1.06	1.54	1.99

“X” means the analyte was below detection limit of 0.01 wt.%.

**Table 2:** Quantitative XRD data (wt.%) for the four precursors and calculated molar Si/Al. See text for explanation of the two quartz phases.

Sample ID	Amorphous Content	Amorphous Molar Si/Al	Quartz1 (SiO_2_)	Quartz2 (SiO_2_)	Mullite (Al_4.7_Si_1.3_O_9.7_)	Hematite (Fe_2_O_3_)	Magnetite (Fe_3_O_4_)	Spinel (MgAl_2_O_4_)	Muscovite (KAl_3_Si_3_O_10_)	Anatase (TiO_2_)
MK1	95.96	1.00	3.28	-	-	-	-	-	-	0.76
MK2	96.57	1.03	1.46	-	-	-	-	-	1.96	-
FA1	52.00	2.39	13	6	25	1	2	1	-	-
FA2	52.00	2.58	20	5	21	2	1	0	-	-

**Table 3:** Oxide composition of amorphous component (wt.%) of the four precursors.

Sample ID	Al_2_O_3_	SiO_2_	CaO	Fe_2_O_3_	K_2_O	Na_2_O	TiO_2_	SiO_2_/Al_2_O_3_	Si/Al
MK1	42.32	50.02	0.10	0.96	0.26	0.07	0.39	1.18	1.04
MK2	41.76	50.92	0.01	0.46	1.35	0.05	0.52	1.22	1.08
FA1	10.20	28.81	1.00	6.95	0.50	0.22	1.83	2.83	2.49
FA2	9.94	30.27	0.67	5.60	0.46	0.24	1.85	3.04	2.69

**Table 4:** Geopolymer formulations showing two fly ash and two metakaolin precursors and target Si/Al, Na/Al, and H/Si.

Precursor	Si/Al	Na/Al	H/Si
MK1	1.8	1.0	5.02
MK1	1.6	0.8	5.34
MK1	1.2	0.6	6.22
MK1	1.7	0.6	5.15
MK2	2.0	1.0	5.62
MK2	1.8	0.9	5.50
MK2	1.6	0.8	5.50
MK2	1.4	0.7	5.50
MK2	1.2	0.6	6.50
FA1	3.0	1.0	4.68
FA1	2.4	1.0	5.48
FA1	1.2	1.0	6.34
FA2	3.0	1.0	3.69
FA2	2.4	1.0	4.27
FA2	1.6	1.0	4.65

**Table 5:** Sample ID is given as two numbers. The first is the Si/Al ratio and the second, in brackets, is the Na/Al ratio. For the strength and peak shift, the number in brackets represents the uncertainty of two standard deviations. A tick in the last column indicates that zeolite was present in the microstructure.

Sample ID	MPa	Peak Shift	Zeolite
FA 1			
1.2 (1)	43 (5)	7.2 (0.3)	✓
2.4 (1)	7 (2)	6.1 (0.2)	
3 (1)	33 (6)	6.9 (0.5)	
FA 2			
1.6 (1)	40 (3)	7.4 (0.3)	✓
2.4 (1)	18 (4)	6.3 (0.2)	✓
3 (1)	70 (9)	8.2 (0.6)	
MK 1			
1.8 (1)	100 (10)	7.6 (0.2)	
1.6 (0.8)	76 (14)	7.2 (0.2)	
1.2 (0.6)	33 (6)	5.9 (0.3)	✓
1.7 (0.6)	4 (1)	5.1 (0.2)	
MK 2			
1.2 (0.6)	3 (1)	5.1 (0.1)	
1.4 (0.7)	57 (9)	6.4 (0.1)	
1.6 (0.8)	61 (8)	6.6 (0.2)	
1.8 (0.9)	55 (6)	6.6 (0.2)	
2.0 (1)	73 (9)	7.0 (0.3)	

## Data Availability

The original contributions presented in this study are included in the article, and further inquiries can be directed to the corresponding author.
